# Circadian metabolism regulates the macrophage inflammatory response

**DOI:** 10.1093/lifemeta/loac037

**Published:** 2022-12-09

**Authors:** Yulong Sun, Wenjiao Jiang, Tiffany Horng

**Affiliations:** School of Life Sciences and Technology, ShanghaiTech University, Shanghai 201210, China; School of Life Sciences and Technology, ShanghaiTech University, Shanghai 201210, China; School of Life Sciences and Technology, ShanghaiTech University, Shanghai 201210, China

**Keywords:** macrophage metabolism, circadian metabolism, circadian rhythm, immunometabolism, circadian immunometabolism, macrophage inflammatory responses

## Abstract

Macrophages are an integral part of the innate immune system and coordinate host defense to microbial infections, as well as shaping the remodeling response after tissue injury. Metabolism is now appreciated to be a powerful and pervasive regulator of the identity and function of macrophages. Upon exposure to microbial ligands, macrophage inflammatory activation and the associated induction of phagocytosis, inflammatory responses, and other host defense activities are supported by dynamic changes to cellular metabolism. Of note, metabolic activity is robustly regulated in a circadian fashion, with many metabolic processes displaying peak activity in one phase of the circadian cycle and trough activity in an antiphase manner. Here, we review recent findings suggesting that circadian metabolism influences macrophage activities and particularly the inflammatory response. First, we summarize macrophage activities known to display time-of-day–dependent variation and their mechanistic basis. Second, we review metabolic processes that have been shown to be rhythmically regulated in macrophages and discuss how such circadian metabolism affects or is likely to affect macrophage activities. Third, we discuss the concept of entrainment of the macrophage clock, and consider how loss of rhythmic regulation of macrophage activities may contribute to pathophysiological conditions like shift work, obesity, and aging. Finally, we propose that circadian metabolism can be used to understand the rationale and mechanistic basis of dynamic regulation of inflammatory responses during infection.

## Introduction

In the last 15 years or so, studies in the burgeoning field of immunometabolism have revealed that cellular metabolism modulates the various activities of macrophages, particularly those of the inflammatory macrophages that orchestrate the induction of immune responses during microbial infection. Macrophage activation by microbial exposure is accompanied by mobilization of metabolism, while perturbation of metabolism can disrupt induction of inflammatory cytokines, microbial killing, and other macrophage activities. For a comprehensive overview of how metabolism modulates macrophage activation, the reader is directed to recent reviews [1, 2]. Here, while particular metabolic pathways and their influence on macrophage activation will be mentioned as necessary, the focus will be on discussing recent studies that have indicated that circadian regulation of metabolism may modulate various activities of inflammatory macrophages.

While macrophage activation and the elaboration of inflammatory responses acutely confer host defense, their sustained induction poses a potential threat to the host. During the course of infection, macrophages are initially activated to induce inflammatory responses and coordinate host defense, but persistent infection triggers macrophages to shift to a tolerant state where they suppress inflammatory responses to limit tissue damage. Therefore, inflammation is a double-edged sword and must be dynamically regulated. Importantly, it has been proposed that anabolic metabolism supports macrophage activation and induction of inflammatory responses, while catabolic metabolism reinforces macrophage tolerance and suppression of inflammatory responses [3, 4].

Metabolism is subject to robust circadian regulation. Some metabolic processes display oscillatory behavior, with peak activity in one phase of the circadian cycle and trough activity occurring in an antiphase manner, while other metabolic processes with bimodal activities may present one activity in a given phase of the circadian cycle and the opposite activity in the other phase of the circadian cycle. For example, during the phase of the circadian cycle when an animal is active (i.e. night for mice and day for humans), hereafter referred to as the active phase, anabolic metabolism, glucose utilization, lipid synthesis, protein synthesis, and AKT-mTOR signaling are upregulated, while during the inactive phase (i.e. day for mice and night for humans), catabolic metabolism, oxidative metabolism, fatty acid oxidation, and autophagy are upregulated. For a comprehensive overview of circadian biology and metabolism, the reader is directed to some recent reviews [5–8]. Here, we will focus on how circadian metabolism influences macrophage activities, such as the regulation of inflammatory responses, a concept that has been previously termed “circadian immunometabolism” [9]. Our review is intended to update recent, informed reviews on this topic [9, 10], and to propose new concepts. For example, we will explore the concept that the macrophage clock should be entrained to allow macrophage activities to be coordinated with other physiological processes, while also highlighting potential entrainment signals. We also propose that circadian metabolism can be used to understand the mechanistic basis and rationale of the dynamic regulation of inflammatory responses during infection. Finally, we will discuss the disease implications of rhythmic oscillations in macrophage activities, and mention disease contexts in which such oscillations are known to be perturbed while highlighting their consequences for pathophysiology.

## Circadian rhythms and biological clocks

In mammals, many biological and physiological processes display circadian rhythmicity, typically of an ~24-h duration that is established directly or indirectly by the Earth’s 24-h rotation on its axis relative to the sun. Circadian rhythms are comprised of alternating active and inactive cycles corresponding roughly to physical day and night periods in diurnal beings, such as humans, and to night and day periods in nocturnal organisms, such as mice. Various biological and physiological processes, such as peak physical activity, reach peak or trough activities at various times within the circadian cycle, or display regular rhythmicity across the circadian cycle, like sleep/wakefulness and feeding/fasting [5]. Circadian rhythmicity allows for synchronization of multiple physiological activities (e.g. sleep and rest should be synchronized with fasting) and for anticipation of regularly occurring physiological activities (e.g. mobilization of some feeding-associated physiological processes early in the active phase in preparation for feeding) [5]. By convention, zeitgeber time (ZT) 0 is the start of the light phase while ZT12 is the start of the dark phase, and unless otherwise stated, studies cited in this review were done in mice, hence ZT0 and ZT12 refer to the start of the inactive phase and active phase, respectively.

**Figure 1:**
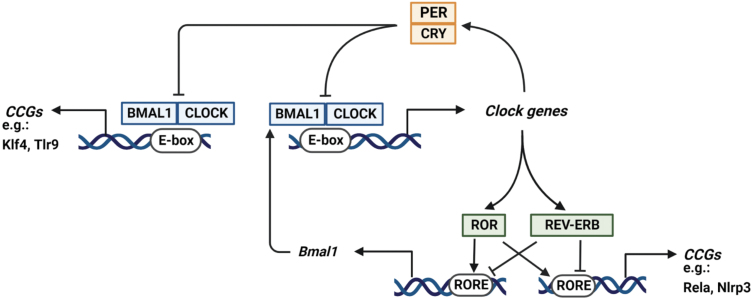
Circadian rhythmicity is established by a molecular clock machinery that consists of several transcriptional complexes whose activities are interlocked in multiple transcriptional-translational feedback loops. A transcriptional heterodimeric complex comprised of BMAL and CLOCK binds to E-boxes in the genes encoding PER and CRY, while the PER-CRY heterodimer competes with BMAL-CLOCK for E-box binding to repress their own genes and eventually initiate a new round of BMAL-CLOCK activity. A second regulatory loop is mediated by BMAL-Clock induction of the genes encoding REV-ERB and ROR, which bind to RORE elements to repress or induce expression of *Bmal1*. The molecular clock machinery also binds to regulatory elements in so-called CCGs, and in macrophages (shown here), and this allows for diurnal rhythms in genes regulating metabolism and immunity (e.g. *Klf4, Tlr9, Rela*, and *Nlrp3*).

Rhythmicity in biological and physiological processes is established by a molecular clock machinery, consisting of several transcriptional complexes whose activities are interlocked in multiple transcriptional-translational feedback loops [6, 7]. At the core of these feedback loops is a transcriptional heterodimeric complex comprised of BMAL (or ARNTL1) and CLOCK, which binds to E-boxes in the genes encoding PER and CRY proteins to induce their expression. After they accumulate, PER and CRY proteins form a heterodimer with transcriptional repressor activity that competes with the BMAL-CLOCK heterodimer for E-box binding, leading to repression of their own genes and eventually a new round of BMAL-CLOCK activity. A second regulatory loop is mediated by BMAL-CLOCK induction of the genes encoding REV-ERB and ROR proteins, which bind to RORE elements to repress or induce expression of the *Bmal* gene. Therefore, a major target of the molecular clock is comprised of genes encoding its own machinery (so-called clock genes). Rhythmicity is inherent within the molecular clock circuitry, while the existence of multiple positive and negative feedback loops ensures that such rhythmicity is robust. In addition to clock genes, the molecular clock machinery also binds to regulatory elements in so-called clock-controlled genes (CCGs) that participate in metabolism, immunity, and other physiological processes. Regulation of CCGs by the molecular clock machinery allows for diurnal rhythmicity in such processes [6, 7] (Fig. 1).

**Figure 2:**
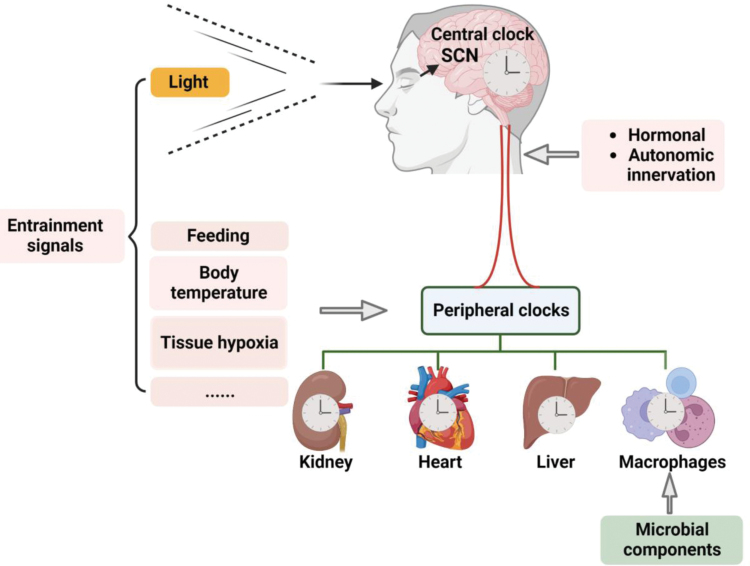
Entrainment signals “set” clock rhythms. Light entrains the central clock in the SCN, which in turn synchronizes peripheral clocks to allow all clocks in the body to “run at the same time”. Although poorly defined, entrainment signals for peripheral clocks are thought to be tissue specific and to include signals emanating from the central clock (hormonal signals and autonomic innervation), as well as feeding-related signals, body temperature, tissue hypoxia, and/or other signals. Microbial components and other signals that drive inflammatory macrophage activation are dominant over steady-state entrainment signals, impinging on the macrophage clock machinery to “reset” its rhythm so that macrophage activities can be prioritized for host defense.

The molecular clock machinery is thought to be present in most cell types, including macrophages and other immune cells. In a healthy animal, all clocks in the body are thought to “run at the same time”, allowing for synchronization of multiple physiological processes. Such clock synchronization is thought to be mediated by a central clock (or master clock) in the suprachiasmatic nucleus (SCN), whose activity is “set” by signals transmitted from the retina upon light sensing. In turn, the master clock synchronizes peripheral clocks (or tissue clocks) through hormonal signals and sympathetic enervation, thereby allowing light-dark cycles to synchronize all clocks in the body [6, 8] (Fig. 2). A more thorough discussion of the regulation of the macrophage clock and the implications for rhythmic oscillations of macrophage functions follows below.

## Circadian regulation of macrophage activities

Diurnal rhythmicity in the mammalian immune system was observed over 6 decades ago, with the finding that mice succumbed more to a night-time challenge with the bacterial component lipopolysaccharide (LPS) than to a day-time challenge [11]. Since then, various activities of the immune system have been shown to exhibit time-of-day–dependent rhythmicity, as previously reviewed [12–14]. Here, the focus will be on circadian regulation of macrophage activities, including induction of inflammatory responses, monocyte trafficking within the body, and phagocytosis. For a discussion of the rationale for circadian regulation of macrophage activities, see Box 1.

**Box 1 *Rationale for circadian regulation of macrophage inflammatory responses***

Multiple non-exclusive hypotheses have been put forth to explain circadian regulation of macrophage activities, and particularly induction of inflammation responses. Animals are more likely to encounter pathogens and become infected at the beginning of the active cycle, as they go out to look for food and engage in social interactions. Thus, peak monocyte recruitment into tissues early during the active cycle anticipates a potential infection at this time. Another rationale that has been proposed has to do with the costs of inducing inflammation: inflammatory response confers host defense at the expense of tissue damage, and perhaps related to this, its induction is energetically and metabolically demanding. Therefore, it may be beneficial to carefully regulate the induction of inflammatory responses, and to coordinate its maximal induction when the animal is most likely to encounter infection and not otherwise [9, 14]. Interestingly, it has been suggested that clock activity may be desynchronized within a population of macrophages, and that this could be a mechanism to regulate inflammatory responses to balance their beneficial vs detrimental effects [15].

### Circadian regulation of monocyte trafficking

**Figure 3:**
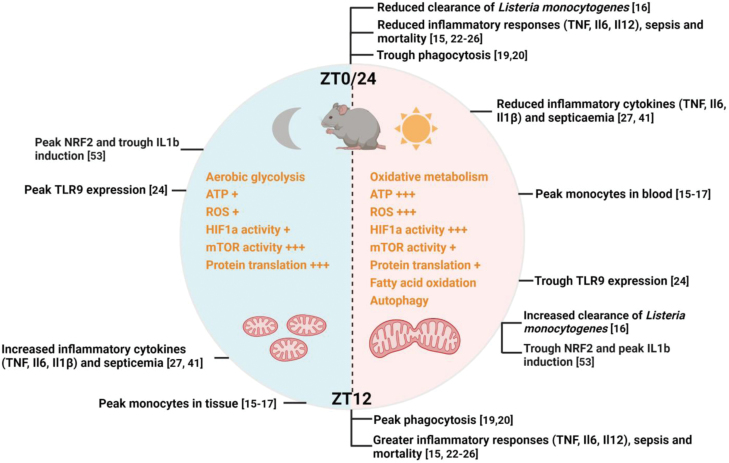
Diurnal oscillations in metabolism influence time-of-day–dependent variation in inflammatory responses and other macrophage activities. In nocturnal mice, the active cycle spans ZT12 to ZT0/24, while the inactive cycle spans ZT0 to ZT12. Rhythmicity in metabolism is highlighted by the prevalence of catabolic metabolism in the inactive cycle and of anabolic metabolism in the active cycle. Inflammatory responses, phagocytosis, and monocyte entry into tissues peak in the active phase, compared to the inactive phase. Citations of the relevant references are indicated in brackets. “+” and “+++” denote relative levels of metabolites and/or relative degrees of activity of various metabolic processes.

Monocytes are cells of the innate immune system that circulate throughout the body at steady state. While monocytes are recruited to sites of infection and tissue damage to coordinate host defense and tissue repair (in some cases concomitant with their differentiation into tissue macrophages), they were recently shown to rhythmically traffic through the blood and peripheral tissues at steady state [16–18]. Monocyte numbers peak in the blood during the inactive phase (ZT5), while their numbers in skeletal muscle peak early in the active phase (ZT13) [16]. It should be noted that while not addressed directly in this study [16], the rhythmicity of monocyte recruitment to the skeletal muscle may facilitate tissue repair following exercise-induced tissue damage that may occur early in the active cycle. Interestingly, rhythmicity was modulated by diurnal expression of adhesion molecules and chemokines in tissue endothelial cells, which in turn was regulated by adrenergic signals delivered by the sympathetic nervous system [16]. In the same study, diurnal expression of adhesion molecules by tissue endothelial cells was also shown to influence the recruitment of neutrophils to the liver during LPS-induced sepsis. Compared to LPS challenge in the inactive cycle, LPS challenge in the active cycle increased expression of the adhesion molecule *Icam* in the hepatic endothelium, leading to enhanced neutrophil influx, and mortality [16]. Collectively, these studies indicate that homeostatic monocyte trafficking is diurnally regulated, with peak tissue recruitment occurring close to the beginning of the active phase, perhaps in anticipation of a need to respond to infection (or exercise-induced tissue damage) (Fig. 3).

The clock machinery has also been shown to directly bind and regulate rhythmic expression of chemokine genes (e.g. *Ccl2* and *Ccl8*) in monocytes and peritoneal macrophages [17, 19]. This could be linked to time-of-day variation in monocyte accumulation in the blood vs tissues, suggesting that the ability of recruited monocytes and tissue resident macrophage to attract more leukocytes and amplify the inflammatory response is modulated by circadian rhythms [17, 19].

### Circadian regulation of phagocytosis

Macrophages are avid scavengers, and their phagocytic activity plays an important role in host defense, resolution of inflammation, and tissue repair. Macrophage phagocytosis of bacterial particles and latex beads has been shown to be circadian regulated, with greater activity at the beginning of the active cycle (ZT12) compared to the beginning of the inactive cycle (ZT0) [20, 21] (Fig. 3). Although diurnal variation in phagocytosis was shown to be not dependent on the macrophage clock in one study [20], another study reported that the macrophage clock negatively regulated phagocytosis in a cell-autonomous manner [21]. In the latter study, genes regulating cell movement and migration were among the top differentially enriched pathways between wildtype and BMAL knockout peritoneal macrophages, and genomic analysis identified genes regulating motility and phagocytosis as direct BMAL targets. Furthermore, deletion of *Bmal* in the myeloid lineage protected mice against pneumococcal infection by conferring greater bacterial clearance, which was attributed to enhanced macrophage motility and bacterial uptake [21]. In a separate study, genes regulating phagocytosis were shown to be controlled by the transcription factor KLF4, whose diurnal expression was dependent on macrophage expression of BMAL [22].

### Circadian regulation of inflammation

Upon pathogen sensing, macrophage production of inflammatory cytokines mobilizes all arms of the immune response, but such production also promotes tissue damage and immunopathology. Since the seminal study of Halberg *et al.* [11], many studies have examined circadian regulation of inflammatory cytokine production. When using an *in vivo* challenge with microbial products (e.g. LPS) or cecal ligation puncture (CLP) as models of sepsis, most studies found that challenge during the active cycle triggered greater inflammatory responses, disease score, and mortality [16, 23–27], compared to challenge during the inactive cycle. Consistently, *ex vivo* analysis of peritoneal macrophages typically showed more robust elaboration of inflammatory cytokines when they were isolated in the active cycle compared to the inactive cycle [23, 24] (Fig. 3).

Rhythmic production of inflammatory cytokines is regulated by the macrophage clock. Myeloid-specific deletion of various clock components (including BMAL, REV-ERBa, and PER) increased production of inflammatory cytokines, particularly in the inactive cycle, indicating that the molecular clock machinery restrains inflammatory responses in this phase of the circadian cycle. Consistently, in response to LPS challenge and CLP, myeloid-specific deletion of clock components led to enhanced sepsis and mortality compared to control mice, a difference that is especially striking in the inactive cycle [23, 24, 27].

What is the underlying basis for the clock-mediated regulation of inflammatory responses? One general mechanism is direct regulation of the genes that encode the components of the signaling pathways that trigger inflammatory gene induction. For example, the gene encoding Toll-like receptor 9 (TLR9), which recognizes hypomethylated CpG DNA in bacterial and viral genomes, was shown to be a direct target of the BMAL-CLOCK complex. In a model of CLP-induced sepsis, oscillatory TLR9 expression was linked to time-of-day–dependent variation in inflammatory cytokine production and mortality [25]. The *Nlrp3* gene was found to be rhythmically regulated in a manner dependent upon direct binding by REV-ERBα. NLRP3 is a component of the inflammasome pathway that regulates maturation of the inflammatory cytokines interleukin (IL)-1b and IL-18, and in the absence of REV-ERBα, dysregulated expression of Nlrp3 leads to increased IL-1b and IL-18 and susceptibility to fulminant hepatitis [28]. NF-κB is a transcriptional master regulator of the inflammatory response, and the *Rela* gene, which encodes the NF-κB subunit p65, was also shown to be directly repressed by REV-ERBα, such that mice with REV-ERBα deletion succumbed more readily to dextran sulfate sodium-induced colitis [29]. It should also be noted that in such regulation of gene expression, clock machinery-mediated effects on chromatin modifications also play a role (reviewed in more detail in [30]). For example, Clock has been shown to have protein acetyltransferase activity and its acetylation of p65 enhances the latter’s DNA-binding activity [31], while REV-ERBα associates with the histone deacetylase HDAC3 to reduce expression of some inflammatory genes [32]. In addition, the clock machinery can influence the activity of various components of the signaling pathways that trigger inflammatory gene induction. For example, CRY was shown to inhibit NF-κB activity via a cAMP-PKA axis, perhaps by via CRY binding to adenyl cyclase to limit cAMP production [33]. Finally, the glucocorticoid receptor (GR) is well-known to powerfully inhibit macrophage inflammatory responses upon glucocorticoid binding. In addition to circadian regulation of glucocorticoid production [34], GR activity is regulated by the clock machinery [35, 36], and in some settings, the ability of glucocorticoids to repress inflammation requires the clock machinery [37]. Therefore, effects on the GR axis may also underpin clock machinery-mediated regulation of inflammatory responses.

A second general mechanism for diurnal regulation of inflammatory responses by the molecular clock centers on metabolism, and will be discussed in detail below. Therefore, inflammatory responses seem to be under multiple levels of circadian control that together coordinate rhythmicity in the inflammatory response, with peak inflammation near the beginning of the active cycle (Fig. 3). Furthermore, loss of clock components generally leads to augmented inflammatory cytokine production.

That inflammatory responses reach zenith levels in the active cycle has implications for various diseases. In many patients, death from sepsis peaks right before the transition to the active cycle [38]. Severity in patients with rheumatoid arthritis reaches an apex in the early morning [39]. In a mouse model of experimental autoimmune encephalomyelitis, disease severity is dependent on the time of day of immunization with the MOG antigen, and the degree of severity is regulated by myeloid cell-expressed BMAL [40]. Microglial activation, which is associated with neurodegenerative diseases, also shows diurnal rhythmicity [41].

### Circadian regulation of host defense

Multiple studies have examined how circadian biology may influence host defense during infections, including those where macrophages play a prominent role. These studies have reported that timing of infection (i.e. during the active vs inactive cycle) can affect levels of inflammatory cytokines, pathogen burden, disease score, and mortality. As discussed above, inducing CLP in the active cycle leads to greater inflammatory responses, sepsis, and mortality compared to induction during the inactive cycle. However, more variability is observed with other infections. For example, infection with *Listeria monocytogenes* at ZT8 compared to ZT0 (late vs early in the inactive cycle) led to better bacterial restriction and higher inflammatory cytokines, perhaps due to more monocyte recruitment to the peritoneal cavity; however, this was also associated with worse survival [17]. In another study, infection with the skin pathogen *Staphylococcus aureus* in the active cycle compared to the inactive cycle was associated with greater production of inflammatory cytokines and less efficient bacterial restriction. This was attributed to reduced production of the rhythmically regulated chemokine CXCL4, which may restrict bacteria by enhancing activation of TLR9 signaling and/or promoting bacterial uptake [21]. In pneumococcal infection, infection during the active phase (ZT15) compared to the inactive phase (ZT3) was associated with increased bacterial burdens in splenic macrophages, even at the earliest time points, which augmented levels of circulating inflammatory cytokines and enhanced septicemia [42]. While myeloid-specific deficiency of clock components exacerbated inflammatory responses and sepsis upon CLP or *L. monocytogenes* infection [17, 27], myeloid-specific *Bmal*-deficient mice controlled bacterial replication better and suffered less weight loss during *S. aureus* infection, which was attributed to enhanced macrophage motility and phagocytosis [21].

Therefore, time-of-day of infection modulates macrophage activities to influence the outcome of pathogen infection. However, the effects of such regulation by the circadian clock may be highly context dependent, being influenced by the nature of host–pathogen interactions and/or of the host defense response, effects of circadian rhythms on the pathogen itself, and other factors.

## Circadian organization of metabolism allows for rhythmic inflammatory responses

Recent studies support the idea that clock regulation of metabolism influences inflammatory responses. *Bmal* knockout macrophages have dysregulated oxidative metabolism. In particular, they have enhanced activity of succinate dehydrogenase and mitochondrial ROS (mtROS) production, which are linked to increased HIF1a-dependent *Il1b* induction [43, 44]. Because ~8%−15% of all genes in the macrophage genome oscillate in a circadian fashion, suggestive of global regulation of the macrophage transcriptome by clock [45, 46], metabolic shifts manifesting in a setting of clock machinery deficiency could reflect direct regulation of the perturbed metabolic pathways by clock, or alternatively, compensatory changes triggered by global disruption of homeostasis. Here, we focus on a discussion and understanding of circadian organization of metabolism as an alternative approach to infer how clock regulation of metabolism can influence rhythmicity in inflammatory responses.

Metabolism is compartmentalized, or organized, in a circadian manner. As mentioned above, the active cycle, which coincides with behavioral activity and feeding, is associated with anabolic metabolism, aerobic glycolysis, mitochondrial fission, and AKT-mTOR signaling, while the inactive cycle, which is contemporaneous with rest and fasting, is characterized by catabolic metabolism, fatty acid oxidation, mitochondrial fusion, and autophagy. Given that metabolism regulates macrophage activities, it is perhaps not surprising that recent studies support the emerging viewpoint that circadian organization of metabolism contributes to diurnal regulation of macrophage inflammatory responses.

One metabolic pathway was consistently found to be rhythmically regulated in macrophages centers on mitochondrial metabolism. Global transcriptomics and proteomics of bone marrow-derived macrophages (BMDMs) subjected to serum shock, which synchronizes the clock *in vitro*, identified oxidative metabolism as being enriched in the inactive cycle and depleted in the active cycle. Both the mitochondrial electron transport chain and the TCA cycle were enriched, and analysis of oxygen consumption using Seahorse extracellular flux analysis corroborated peak oxidative metabolism in the inactive cycle. Conversely, aerobic glycolysis, which is often regulated reciprocally to oxidative metabolism, reached apex levels in the active cycle [45]. Mitochondrial oxidative metabolism is closely linked to mitochondrial fission and fusion, which were also found to be regulated in a rhythmic manner [45, 47] (Fig. 3). Such rhythmicity in mitochondrial metabolism is likely to be regulated by BMAL. In hepatocytes, diurnal oscillations in oxidative metabolism were dependent on BMAL [48], and *Bmal* knockout BMDMs were shown to have increased glucose oxidation and oxidative metabolism, as well as enhanced mtROS production [43, 44].

How rhythmicity in mitochondrial metabolism influences macrophage activities remains less characterized. Using pharmacological inhibition of mitochondrial ATP synthase, Hurley *et al*. suggested that rhythmic oxidative metabolism may modulate phagocytosis of the yeast particle [45]. Importantly, many studies in the field of immunometabolism have shown that mitochondria metabolism can regulate macrophage activities, in particular induction of inflammatory responses [49]. For example, aerobic glycolysis rather than oxidative metabolism is often linked to induction of inflammatory responses, correlating with peak aerobic glycolysis in the active cycle, but peak oxidative metabolism in the inactive cycle. Similarly, fizzed mitochondria have been linked to induction of inflammatory responses [50, 51]. Finally, the capacity to dynamically regulate oxidative metabolism in the inactive cycle (specifically to enforce a shift from increased to decreased oxidative metabolism) may facilitate a shift from induction to suppression of inflammatory responses by limiting oxidative metabolism-dependent production of Ac-CoA and histone acetylation at inflammatory genes [52]. Therefore, circadian organization of mitochondrial metabolism likely contributes to diurnal induction of inflammatory responses and likely other macrophage activities. Consistent with this notion, disruption of oxidative metabolism in *Bmal* knockout BMDMs was associated with enhanced induction of the inflammatory cytokine gene *Il1b*, via activation of the transcription factor HIF-1a by enhanced mtROS production [43, 44].

Recent studies have indicated that redox metabolism may be circadian regulated in macrophages [44, 53]. Specifically, ROS levels are high in the inactive cycle (ZT8) relative to the active cycle (ZT20), attributed to BMAL regulation of NRF2, a transcription factor that controls the expression of ROS scavenging enzymes and other aspects of the antioxidant response. Importantly, this rhythmic NRF2-ROS axis influences HIF1a activity to regulate the induction of *Il1b*. Therefore, oscillatory regulation of NRF2 allows for ROS scavenging and suppression of inflammatory responses in the inactive cycle [54] (Fig. 3). Because ROS levels modulate many other macrophage activities, including antimicrobial killing and phagocytosis, such a rhythmic NRF2-ROS axis is likely to have a pervasive influence on macrophage functions [53].

Protein metabolism is subject to circadian regulation in macrophages [45]. First, proteomic and transcriptomic profiling around the clock indicates that proteins accumulate in a rhythmic manner, and for many proteins, such accumulation is heavily influenced by post-transcriptional events rather than being strictly regulated by transcription. Therefore, rhythmic post-transcriptional and translational regulation may play an important role in macrophage biology. Second, macrophage protein translation peaks in the active phase [45]. Although the consequence of such peak translation was not addressed, it is likely to contribute to maximal induction of inflammatory responses given that macrophage activation in response to microbial exposure is known to be accompanied by a robust upregulation of protein synthesis. Mechanistically, such peak translation is likely to be regulated by time-of-day–dependent variations in mTOR activity. mTOR activity is known to drive cap-dependent translation and ribosome biogenesis to promote protein synthesis [55], and displays oscillatory behavior in the liver, reaching an apex in the active phase [56]. Finally, macrophage protein degradation was found to be subject to circadian regulation [45]. Maximal degradation of electron transport chain components and TCA cycle enzymes in the active cycle allows for accumulation of these proteins antiphase in the inactive cycle, which appears to contribute to the peak oxidative metabolism that occurred in the inactive cycle discussed above.

In summary, available evidence indicates that circadian changes to metabolism can modulate diurnal changes in macrophage inflammatory responses. The evidence so far is consistent with the general framework that anabolic metabolism and a high metabolic state, characterized by aerobic glycolysis, mTOR activity, protein translation, and mitochondria fission, reinforce induction of inflammatory responses. Conversely, catabolic metabolism and a low metabolic state, associated with oxidative metabolism, mitochondria fusion, and antioxidant responses, support suppression of inflammatory responses. Many other metabolic processes are known to be regulated in a circadian manner in other contexts, including fatty acid oxidation and synthesis, NAD metabolism, and autophagy [5, 57]. Some of these processes have already been reported to modulate macrophage activities (although not circadian macrophage biology). Importantly, according to the framework that we have proposed, those processes not already known to do so are likely to modulate macrophage inflammatory responses in a predictable fashion. Thus, a better understanding of circadian metabolism in macrophages is likely to inform the rationale and etiology of metabolic modulation of macrophage inflammatory responses.

## Entraining macrophage activities

Within a healthy animal, all clocks in the body are thought to be synchronized, enabling coordination across physiological systems. For example, peak inflammatory responses occur around the beginning of the active cycle, indicating coordination of the macrophage clock with other clocks in the body, including those that regulate sleep/wakefulness and physical activity. This raises the important question of what is responsible for “setting” rhythms in the macrophage clock?

As mentioned above, light acts as an entrainment signal (sometimes also referred to as zeitberger) for the master clock, which then synchronizes peripheral clocks via a combination of direct and indirect signals, including hormonal signals, autonomic innervation, feeding-related signals, body temperature, and tissue hypoxia [6, 8]. Entrainment signals act in a cell type-specific fashion, and establish rhythmicity of peripheral clocks by influencing the expression and/or activity of various clock components. Light is the dominant entrainment signal for the master clock, but acting directly has modest effects on most peripheral clocks. In contrast, feeding rather than light is the primary entrainment signal for the liver, consistent with the central role of the tissue in metabolism [58–60]. In macrophages, little is known regarding what may be the relevant entrainment signal(s). As mentioned above, monocyte trafficking appears to be regulated by endothelial expression of adhesion molecules downstream of adrenergic signaling [16], but it seems likely that at least some other macrophage/monocyte activities may not be regulated by the same sympathetic nervous system-endothelial cell axis. Glucocorticoids are produced by the adrenal cortex in a diurnal manner and is thought to act as an entrainment signal in some tissues [6], and macrophage inflammatory responses are known to be modulated by GR [61]; but adrenalectomy of mice to ablate glucocorticoid production does not affect time-of-day–dependent production of inflammatory cytokines in LPS-stimulated macrophages [46]. Interestingly, feeding has been shown to entrain inflammatory responses. Restricting feeding to the inactive cycle was sufficient to reverse the inducibility of the inflammatory response, such that it was more inducible by LPS challenge in the inactive cycle than in the active cycle [62]. Such entrainment of the inflammatory response to some feeding-associated cue may be intuitive, given that metabolism is an important regulator of the inflammatory response (Fig. 2).

An issue related to entrainment of the macrophage clock is how exposure to the microbial components and associated signals that drive inflammatory macrophage activation affects clock activity. Available evidence indicates that signaling pathways downstream of these signals impinge on various components of the clock machinery to “reset” the period, phase, and amplitude of clock oscillations. For example, exposure to LPS and other microbial signals suppressed the oscillatory amplitude of and induced a phase shift in PER2, which appeared to be mediated in part by LPS-induced increases in ROS [63, 64]. Similarly, LPS challenge of mice led to a shortening of the lung circadian cycle from ~24 to ~20 h [65]. Mechanistically, LPS signaling impinges on the clock at least in part by inducing the expression of mir-155, which acts to downregulate levels of *Bmal* transcript [24]. Intuitively, microbial signals should be dominant over steady-state entrainment signals, triggering a resetting of clock activity that alters rhythms in the expression of CCGs, including genes regulating metabolism and activities associated with inflammatory macrophage activation. How such altered rhythms affect macrophage activation and host defense remains poorly characterized, but may be an interesting avenue of investigation in future studies.

## Disease implications: when circadian regulation of inflammation goes awry

There are abundant epidemiological data that direct disruption to circadian rhythms in shift work and jet lag predisposes to obesity, cardiovascular diseases, autoimmune diseases, and cancer [66–71]. Many of these diseases are driven by pathophysiological inflammation, suggesting that disruption of the macrophage clock by shift work and jet lag likely contributes to disease pathogenesis, in addition to loss of circadian rhythms in the parenchymal compartment. In support of this notion, chronic disruption of circadian timing abrogates clock rhythmicity in peritoneal macrophages, increases severity of LPS-induced septic shock [72], and enhances macrophage accumulation in various tissues [73]. Chronic dim light exposure in rats perturbs the diurnal variation of monocyte numbers in circulation [74] and elevates circulating monocyte numbers in shift workers [75, 76].

There is also evidence that the macrophage clock can become disrupted in other pathophysiological settings. High-fat diet feeding, which disrupts the hepatic clock and some other tissue clocks, perturbs the macrophage clock, which is linked to increased macrophage inflammatory activation and systemic insulin resistance [77]. A recent study found that aging disrupts the macrophage clock [22]. Aging is known to attenuate the robustness of the molecular clock, which is thought to contribute directly to the aging-associated loss of metabolic homeostasis and general tissue homeostasis. In macrophages, aging does not precipitate changes in the expression and oscillation of the core clock machinery, but leads to loss of rhythmicity in some CCGs, dampens diurnal variations in monocyte trafficking and macrophage phagocytosis, and enhances susceptibility to endotoxin induced mortality [22]. The transcription factor KLF4, rhythmic in macrophages from young but not aged mice, regulates the expression of genes encoding phagocytosis and is implicated in such loss of oscillatory phagocytosis. Interestingly, a KLF4 variant was identified in the human population that confers age-dependent susceptibility to death from bacterial infection. Therefore, aging is associated with perturbed rhythmicity of some macrophage clock activities, leading to increased susceptibility to septic shock but impaired host defense [22].

What are the therapeutic implications of circadian regulation of macrophage inflammatory responses? REV-ERBα, which represses *Bmal* expression in the negative feedback loop of the clock circuitry, has been explored as a therapeutic target. Notably, administration of a synthetic REV-ERBα agonist that reduces inflammatory gene expression *in vitro* attenuates LPS-induced acute lung injury and inflammation in mice [23, 78]. As a nuclear receptor (and thus readily druggable), REV-ERBα could be pursued further as a potential therapeutic target in settings of excessive inflammation [79]. Similarly, GR agonists, which powerfully inhibit induction of inflammatory responses, have been used therapeutically in rheumatoid arthritis and other settings of macrophage-mediated pathophysiological inflammation, but with some notable side-effects (e.g. long-term immunosuppression and metabolic dysregulation) [80]. Small molecular modulators of the clock machinery are another attractive target. Small molecule activators of Cry proteins have been identified [81], as well as an ROR agonist with clock amplitude-enhancing effects [82]. Given that the macrophage clock amplitude is dampened in obesity, aging, and chronic shift work, the aforementioned ROR agonist could be explored in restoration of clock rhythmicity in various disease settings. Finally, manipulating metabolism as a way to alter circadian regulation of inflammatory responses is another appealing option, but the ability to target macrophage-specific metabolic pathways may be advisable to avoid general effects of perturbing ubiquitous metabolic pathways. Altogether, continued efforts towards a better understanding of how circadian rhythms modulate the inflammatory response will yield more insight into therapeutic control of many diseases where macrophage-derived inflammatory responses predispose to pathogenesis.

## Summary and future outlook

Studies in the immunometabolism field have firmly established metabolism as a key regulator of macrophage activities, and particularly in the elaboration of inflammatory responses. As discussed in this review, circadian metabolism influences time-of-day–dependent changes in macrophage activities. Mitochondrial metabolism (including oxidative metabolism and mitochondrial dynamics), ROS metabolism, and protein translation oscillate in macrophages, contributing to rhythmic changes in production of inflammatory cytokines and other activities. Future studies in this area are likely to uncover additional nodes by which circadian metabolism regulates macrophage activities.

Inflammatory responses are dynamically modulated during infection, being induced acutely after infection but suppressed during persistent infection (e.g. resulting from genetic immunodeficiency of the host or infection with high pathogen burden). Such modulation of the inflammatory response balances the needs of host defense with the potential for tissue damage, and importantly, recent studies indicate an underpinning role for dynamic shifts to metabolism. Here, we propose that circadian metabolism with its inherent rhythmicity may play a role in the dynamic regulation of inflammatory responses. Future studies addressing how circadian metabolism influences macrophage biology may shed further light on the rationale and mechanistic basis for the dynamic regulation of macrophage inflammatory responses, of broad relevance to the many infectious diseases where runaway inflammation poses a latent threat.

The relevant entrainment signals for the macrophage clock remains unknown, but as mentioned above, coordination of the macrophage clock with other tissue clocks and the master clock is likely to be important for maintaining tissue homeostasis while being able to optimally mobilize host defense. Shift work, estimated to represent ~15% of the working population in some countries, disrupts the macrophage clock, likely contributing to the enhanced predisposition to obesity and metabolic syndrome in this population. Similarly, dysregulation of the macrophage clock occurs in common conditions, including obesity and aging, where increased macrophage-derived inflammation likely influences pathophysiology. Thus, elucidating entrainment of the macrophage clock, as well as clock-regulated activities in macrophages, should pave the way towards a better understanding of the role of macrophages in coordinating tissue homeostasis, and conversely, how their disruption leads to disease.
